# Design of High Temperature Ti-Pd-Cr Shape Memory Alloys with Small Thermal Hysteresis

**DOI:** 10.1038/srep28244

**Published:** 2016-06-22

**Authors:** Deqing Xue, Ruihao Yuan, Yumei Zhou, Dezhen Xue, Turab Lookman, Guojun Zhang, Xiangdong Ding, Jun Sun

**Affiliations:** 1 State Key Laboratory for Mechanical Behavior of Materials, Xi’an Jiaotong University, Xi’an 710049, China; 2 School of Materials Science and Engineering, Xi’an University of Technology, Xi’an 710048, China; 3 Theoretical Division, Los Alamos National Laboratory, Los Alamos, New Mexico 87545, USA

## Abstract

The large thermal hysteresis (ΔT) during the temperature induced martensitic transformation is a major obstacle to the functional stability of shape memory alloys (SMAs), especially for high temperature applications. We propose a design strategy for finding SMAs with small thermal hysteresis. That is, a small ΔT can be achieved in the compositional crossover region between two different martensitic transformations with opposite positive and negative changes in electrical resistance at the transformation temperature. We demonstrate this for a high temperature ternary Ti-Pd-Cr SMA by achieving both a small ΔT and high transformation temperature. We propose two possible underlying physics governing the reduction in ΔT. One is that the interfacial strain is accommodated at the austenite/martensite interface via coexistence of B19 and 9R martensites. The other is that one of transformation eigenvalues equal to 1, i.e., *λ*_2_ = 1, indicating a perfect coherent interface between austenite and martensite. Our results are not limited to Ti-Pd-Cr SMAs but potentially provide a strategy for searching for SMAs with small thermal hysteresis.

Shape memory alloys (SMAs) undergo a reversible martensitic phase transformation from the high symmetry austenite (A) to low symmetry martensite (M) phase upon the influence of temperature or stress field, giving rise to the shape memory effect (SME) and superelasticity (SE), respectively^1^,^2^. Both thermally induced and mechanically induced martensitic transformations involve hysteresis, i.e., the forward and reverse martensitic phase transformations do not coincide^3^,^4^. The hysteresis is the macroscopic manifestation of the dissipated energy during a phase transformation and it is generally considered to originate largely from the strain incompatibility at the A/M interface, which gives rise to an energy barrier for the phase transformation^3^,^5^,^6^. During the cyclic thermal or mechanical martensitic phase transformation, strain incompatibility introduces several irreversible processes, such as the generation of dislocations and microcracks, resulting in serious fatigue^7^,^8^,^9^. The fatigue degrades physical, mechanical properties of SMAs, especially the SME and SE, and finally leads to failure^7^. Therefore, the “reversibility”, the ability to pass back and forth through the phase transformation many times without degradation of properties, is critical and extensive research has focused on reducing hysteresis in order to improve the “reversibility” of SMAs^3^,^4^,^10^,^11^,^12^,^13^.

In searching for thermoelastic SMAs with small thermal hysteresis (ΔT), several different methodologies have been utilized. Experimentally, combinatorial synthesis of SMA thin films has been employed to screen the various compositions and select the best candidates^10^,^11^,^13^,^14^. Very recently, an adaptive design strategy based on machine learning algorithms has been shown to effectively explore the compositional space to identify alloys with very small hysteresis^15^. Theoretically, the geometrically non-linear theory of martensite (GNLTM) has been very useful in guiding the search for better alloys^5^,^10^. The martensitic transformation can be described by the symmetric transformation matrix *U*, which maps the martensite lattice to the austenite lattice^16^,^17^. The ordered eigenvalues of *U*, *λ*_1_ ≤ *λ*_2_ ≤ *λ*_3_, represent the presence of an invariant habit plane between austenite and martensite^16^,^17^. The GNLTM provides the constraint, *λ*_2_ = 1, as means to reduce ΔT, so that there is a perfect coherent interface (unstressed and untwinned) between austenite and martensite^5^. Coupled with a combinatorial synthesis method, the GNLTM has led to the discovery of Ti-Ni-Cu and Ti-Ni-Cu-Pd systems with very small ΔT^10^,^14^. The correlation between *λ*_2_ = 1 and Δ*T* has been confirmed for Ti-Ni-Au, Ti-Ni-Pt and Ti-Ni-Pd ternary systems by various authors^5^,^18^,^19^,^20^.

However, the use of GNLTM theory to design new SMAs requires *a priori* knowledge of crystal symmetry and lattice parameters, or the relationship between lattice parameters and alloying elements so that *λ*_2_ can be evaluated in advance. The access of those information beforehand requires a lot of experimental efforts, especially for multicomponent systems. Thus, a simple strategy that can use the knowledge available to guide the design of SMAs with small ΔT is highly desireable.

In the present study, we propose that a compositional crossover region between two different types of martensitic transformations can give rise to small ΔT. Figure 1 shows our idea for designing new SMAs with small thermal hysteresis. Upon cooling, the martensitic transformation in some SMAs is accompanied by an increase in electrical resistance across the transformation from A to M (shown in Fig. 1(a) by +Δ*R*), whereas in other SMAs the martensitic transformation is accompanied by a decrease in resistance at the martensitic transformation, as denoted in Fig. 1(b) by −Δ*R*. Different martensites can be characterized by the distinct behavior they show with respect to Δ*R*. A +Δ*R* is due to the higher electrical resistance of M over A; and −Δ*R* indicates that M has a lower resistance than A. Examples of martensites with +Δ*R* include Ti_50_Ni_50_ (aged), Ti_50_Ni_50−*x*_Cu_*x*_, Ti_50_Ni_50−*x*_Fe_*x*_, Ti_50_Pd_50−*x*_Cr_*x*_ (x > 5%*at*.), whereas those with −Δ*R* include Ti_50_Ni_50_, Ti_50_Ni_50−*x*_Pd_*x*_, Ti_50_Pd_50−*x*_Cr_*x*_ (x < 4%*at*.)^21^. It usually is the case that martensitic transformations with +Δ*R* can gradually change to those with −Δ*R* by either defect doping or thermal treatment^21^,^22^. Therefore, we suggest that there exists a crossover so that a martensitic transformation can be accompanied without any change in resistance, i.e., Δ*R* = 0, as shown in Fig. 1(d). In another words, with Δ*R* continuously or monotonically varying with either defect doping or thermal treatment, there must exist a point or regime where Δ*R* = 0. In such a case, a martensitic transformation occurs without any change in resistance both on cooling and on heating, thus the thermal hysteresis would be expected to be zero (Fig. 1(c)).

We note that the energy dissipation (ΔT here) is much more severe in high temperature SMAs and as the transformation temperature increases, the thermal hysteresis should also increase dramatically. For example, *Ti*_50.0_*Ni*_50.0_ possesses a martensitic transformation temperature of 363 K and a ΔT of 30 K, whereas the ternary alloys Ti-Ni-Hf and Ti-Ni-Zr have higher transformation temperatures around 473 K and a much larger ΔT of 60 K. In the present study, we note that the Ti-Pd SMA system has a high martensitic transformation temperature, and more importantly, its martensitic transformation has the desirable feature of +Δ*R* and −Δ*R* depending on the different concentrations of defects. We thus demonstrate the key concept shown in Fig. 1 for the Ti-Pd-Cr SMAs by accomplishing both a small ΔT and a high transformation temperature. The functional stability from differential scanning calorimetry of our best alloy *Ti*_50_*Pd*_45.3_*Cr*_4.7_ upon thermal cycling also significantly improved compared to the archetypal SMA *Ti*_50.0_*Ni*_50.0_. Our finding thus provides a straightforward strategy for searching for SMAs with small thermal hysteresis and high reversibility.

## Results

To verify our strategy, we fabricated the *Ti*_50_*Pd*_50−*x*_*Cr*_*x*_ system between 4 *at*.% and 5 *at*.% Cr. All the samples were fabricated in the present study under the exact same conditions to avoid the possible effects from microstructure and defects. We first checked the crystal structure of our samples. Typical X-ray diffraction profiles of all the samples are shown in Fig. 2(a). It is clear that all the diffraction peaks can be indexed by B19 martensite. However, it is also shown in the literature that the TiPd samples with high defect concentration, such as 5 *at*.% Cr, may possess 9R martensite structure as well^23^. We further calculated the unit cell volume from our X-ray diffraction profiles and the result is shown in Fig. 2(b). A minimum unit cell volume occurs at 4.6 *at*.% Cr, indicating something special happens and will be discussed in the discussion part.

**Figure 1:**
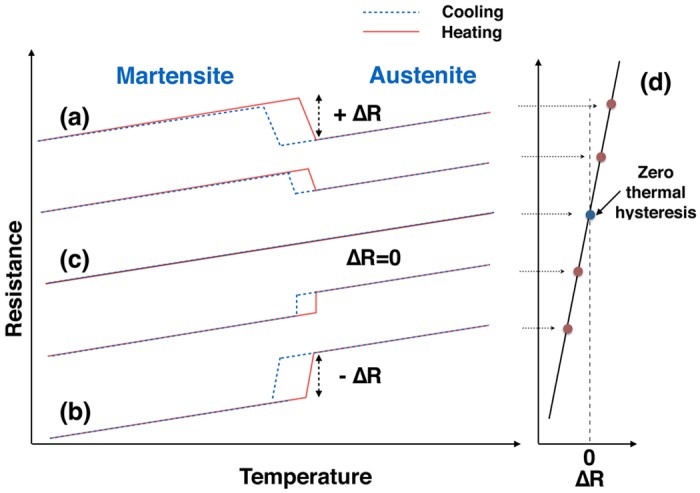
Schematic of the search strategy for SMAs with small thermal hysteresis. A martensitic transformation can result in (a) an increase of resistance (+Δ*R*) from A to M, or (b) a drop in resistance −Δ*R*, upon cooling. Therefore, (d) a crossover with Δ*R* = 0 can take place if Δ*R* varies with either defect doping or thermal treatment, (c) At the crossover condition, Δ*R* = 0, where the martensitic transformation can occur without any change in resistance upon cooling and heating, the alloy is presumed to have thermal hysteresis equal to zero.

Figure 3 shows the resistance versus temperature (R(T)) curves of a number of compositions for the *Ti*_50_*Pd*_50−*x*_*Cr*_*x*_ system between 4 *at*.% and 5 *at*.% Cr. R(T) curves with −Δ*R* can be found in samples with Cr concentration near 4 *at*.%, and R(T) curves with +Δ*R* are observed in samples with Cr concentration near 5 *at*.%. The former −Δ*R* situation corresponds to the B2 to B19 martensitic transformation that is typically found in *Ti*_50_*Pd*_50_ alloy, whereas the latter +Δ*R* is due to the B2 to 9R martensitic transformation which appears after heavily doping the *Ti*_50_*Pd*_50_ alloy with Cr, V, or Mn^24^,^25^. For Cr concentration between 4.5 *at*.% and 4.7 *at*.%, the R(T) curves do not possess the usual “S” shape at the transformation temperature, instead, show a tendency of flattening and merging together. Such evolution of Δ*R* from a positive value through almost zero to a negative value is exactly the situation expected in Fig. 1. According to the concept proposed in Fig. 1, alloys within this crossover composition range potentially possess very small Δ*T*.

Figure 4(a) shows how the thermal hysteresis ΔT varies with Cr concentration in *Ti*_50_*Pd*_50−*x*_*Cr*_*x*_. The ΔT is calculated using 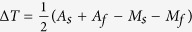, where *M*_*s*_ and *M*_*f*_ are the martensitic transformation start and finish temperatures, and *A*_*s*_ and *A*_*f*_ are the reverse transformation start and finish temperatures. These start and finish temperatures are determined using the tangent method as shown in the inset of Fig. 4(a). The ΔT is large for Cr concentration around 4.0 *at*.% with −Δ*R*, and then decreases with increasing Cr concentration. It reaches a minimum at Cr concentration of 4.7 *at*.%, which is just within the composition crossover region mentioned above. ΔT then increases again for Cr concentration around 5.0 *at*.% with +Δ*R*. We also determined ΔT from DSC measurements via endothermic and exothermic peaks, as shown in Fig. 4(b). The tangent method was employed to obtain *A*_*s*_, *A*_*f*_, *M*_*s*_ and *M*_*f*_; and ΔT was calculated using the same equation as for the R(T) curves. The ΔT from DSC measurements behaves identically to that obtained from R(T) curves, emphasizing the consistency of our approach. In addition to the tangent method, we also calculated ΔT from the exothermal/endothermic peak temperatures, Δ*T* = *T*_*exo*_ − *T*_*end*_, as shown in the left inset to Fig. 4(b). For this DSC measurement, we still see the characteristic “V” curve but with the smallest ΔT at 4.6 *at*.%, which is still in the crossover composition region. The “V” behavior in Fig. 4 is an experimental validation of our design recipe of Fig. 1.

## Discussion

**Figure 2:**
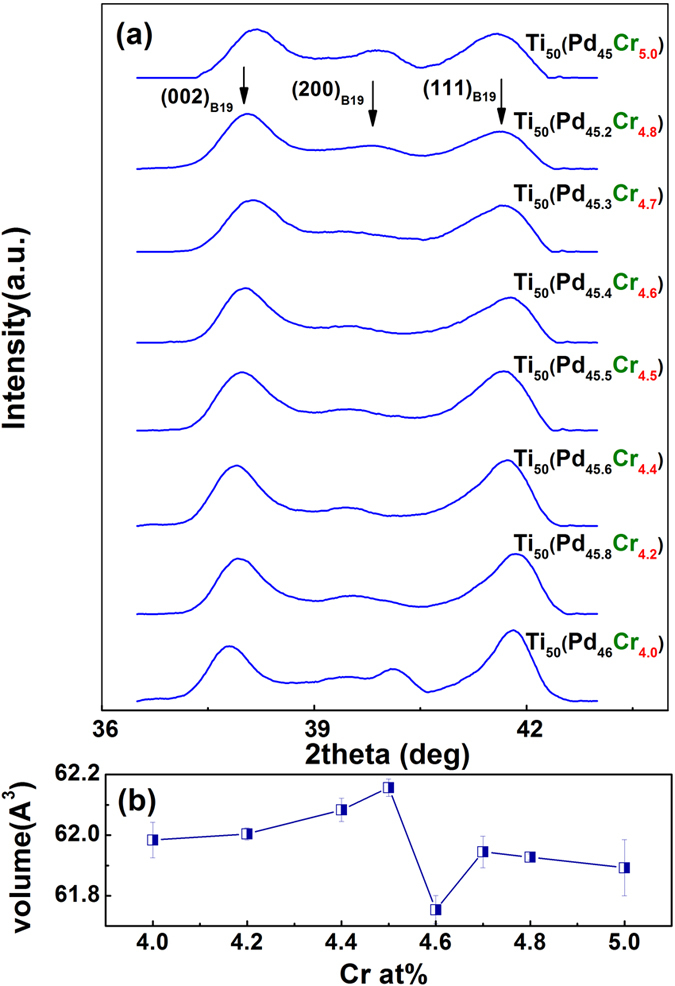
Crystal structure of *Ti*_50_*Pd*_50−*x*_*Cr*_*x*_ SMAs system between 4 *at.*% Cr and 5 *at.*% Cr. (**a**) X-ray diffraction profiles show three typical peaks that can be indexed as (002)_*B*19_, (200)_*B*19_ and (111)_*B*19_. (**b**) the calculated unit cell volume of samples with different Cr concentrations. A minimum of unit cell volume can be found at the 4.6 *at*.% Cr sample. The error bars were added by the fitting errors of the Bragg peaks.

**Figure 3:**
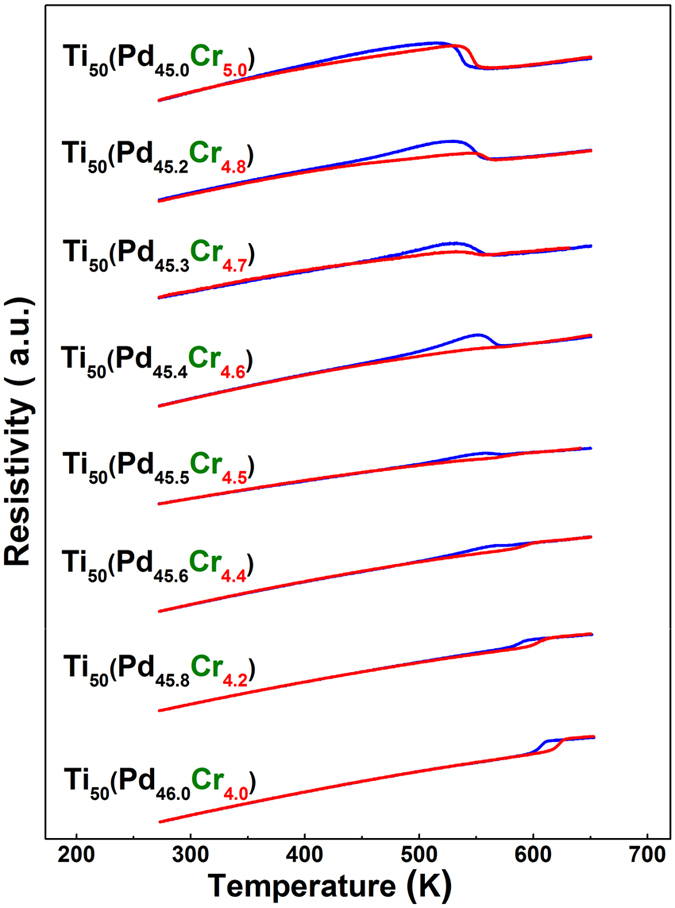
Experimental validation of our design recipe in *Ti*_50_*Pd*_50−*x*_*Cr*_*x*_ SMAs. R(T) curves for *Ti*_50_*Pd*_50−*x*_*Cr*_*x*_ with different Cr concentrations are shown. The red line indicates the heating process while the blue one is the cooling curve. The −Δ*R* occurs at *x* around 4 *at*.%, and the +Δ*R* occurs at *x* around 5 *at*.%. Crossover region between 4.5*at*.% and 4.7*at*.% can give rise to narrow thermal hysteresis.

**Figure 4:**
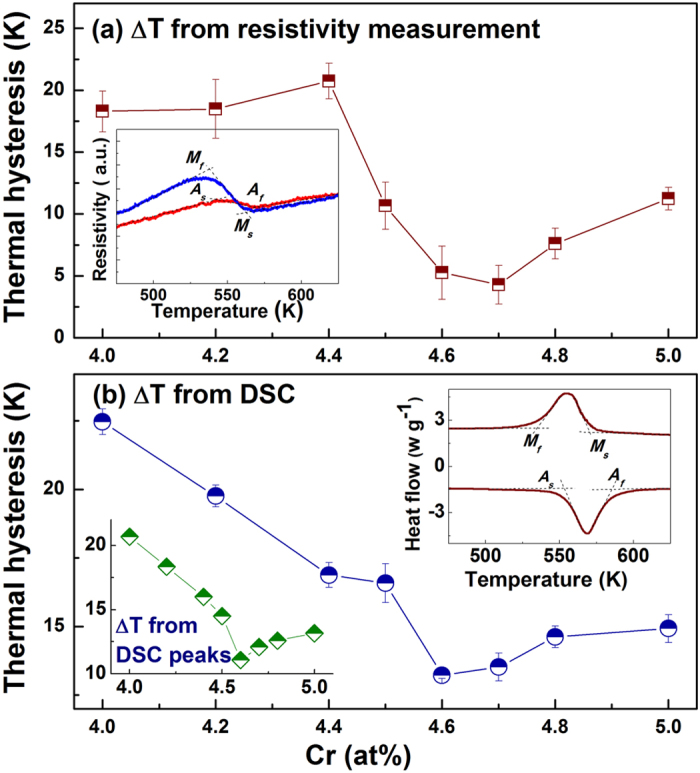
The behavior of the thermal hysteresis Δ*T* as a function of concentration is consistent with our notion that small ΔT occurs in the crossover region for *Ti*_50_*Pd*_50−*x*_*Cr*_*x*_. (**a**) 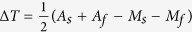 from R(T) measurements, where *A*_*s*_, *A*_*f*_, *M*_*s*_ and *M*_*f*_ are determined by the tangent method (inset). (**b**) 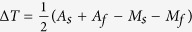 from DSC measurements, where *A*_*s*_, *A*_*f*_, *M*_*s*_ and *M*_*f*_ are determined using the tangent method (right corner inset). The ΔT directly determined by exothermal/endothermic peak temperatures are shown in the left corner inset, revealing the same behavior. The error bars were determined by choosing the start and finish temperatures (*A*_*s*_, *A*_*f*_, *M*_*s*_ and *M*_*f*_) for several times employing the tangent method.

It is known that +Δ*R* and −Δ*R* correspond to the B2-B19 and B2-9R martensitic transformations, respectively. The optimized compositions just sit in the crossover region between B2-B19 and B2-9R transformations. Thus the optimal, smallest ΔT is likely to be due to instabilities associated with the three phases which include B2, B19 and 9R. At the phase transformation temperature in the crossover region, the three phases can be degenerate in energy, allowing the possibility of multiple transformation pathways. Most likely, the preferred pathway in the energy landscape is one that include a saddle point between the maxima of B19 and 9R. Microstructurally, the saddle point and pathway would correspond to coexisting B19 and 9R martensite at the A/M interface so that the strain at the A/M interface is easily accommodated via both B19 twins and 9R twins. However, for direct B2-B19 and B2-9R transformations, energy barriers need to be overcome for the phase transformations to take place. This is accompanied by larger strains at the habit planes for B2-B19 and B2-9R, resulting in larger ΔT. This explanation is consistent with the initial decrease in ΔT from a larger value to a fairly small value followed by an increase again to a relatively large value. Another possible physical origin relies on the eigenvalues of transformation strain matrix. Due to the strong correlation between *λ*_2_ = 1 and small Δ*T*, it is also natural to expect that the samples with smallest Δ*T*, i.e., 4.6 *at*.% Cr and 4.7 *at*.% Cr, may possess *λ*_2_ = 1. The abnormal unit cell volume change within 4 *at*.% and 5 *at*.% Cr shown in Fig. 2(b) may be supportive to this argument, as the *λ*_2_ is closely related with the lattice parameters of martensite. Consequently, a perfect coherent interface (unstressed and untwinned) between austenite and martensite should be present in those TiPd samples with smallest Δ*T*. Although we provided two possible explanations for the low thermal hysteresis of our newly found alloy, the exact underlying mechanism still needs to be clarified in the future work.

**Figure 5:**
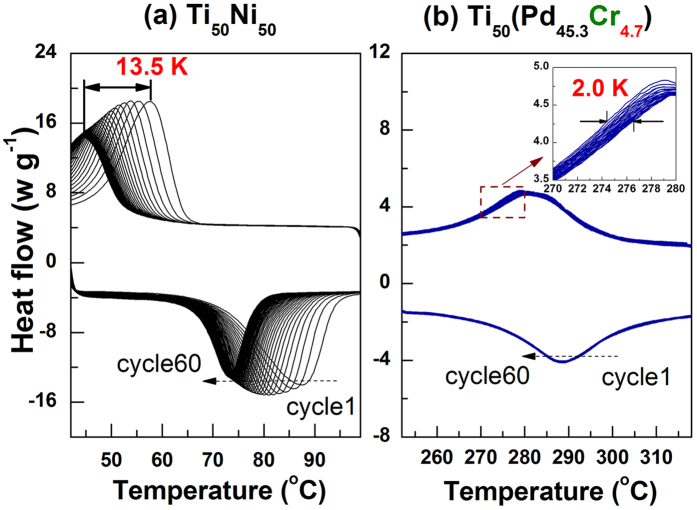
Functional fatigue behavior of bulk alloys. Compilation of 60 DSC cycles plotted for (**a**) *Ti*_50_*Ni*_50_, (**b**) *Ti*_50_*Pd*_45.3_*Cr*_4.7_. The inset in (**b**) enlarges the shift of the DSC curves for *Ti*_50_*Pd*_45.3_*Cr*_4.7_. The shift in *Ti*_50_*Ni*_50_ of 13.5 K is much larger than that in *Ti*_50_*Ni*_50_ of 2.0 K, indicating a significant improvement in functional fatigue.

The transformation hysteresis is known to be directly correlated with fatigue properties for SMAs. A smaller ΔT means that strain compatibility is satisfied relatively easily at the A/M interface and results in improved functional fatigue properties for SMAs cycled thermally. Thus, we investigated the functional stability of Ti_50_Ni_50_ and our best alloy, *Ti*_50_*Pd*_45.3_*Cr*_4.7_, by thermal cycling using DSC. Figure 5 shows a compilation of 60 DSC cycles for the binary and ternary alloys. The shift of the DSC curves (transformation temperature) is about 13.5 K upon thermal cycling 60 times, as shown in Fig. 5(a). In contrast, the shift upon cycling is much less visible for our *Ti*_50_*Pd*_45.3_*Cr*_4.7_ alloy (Fig. 5), and the inset highlights that the shift is only 2 K. Thus, we significantly improve the functional fatigue properties of our *Ti*_50_*Pd*_45.3_*Cr*_4.7_ alloy via the design strategy in Fig. 1.

**Figure 6:**
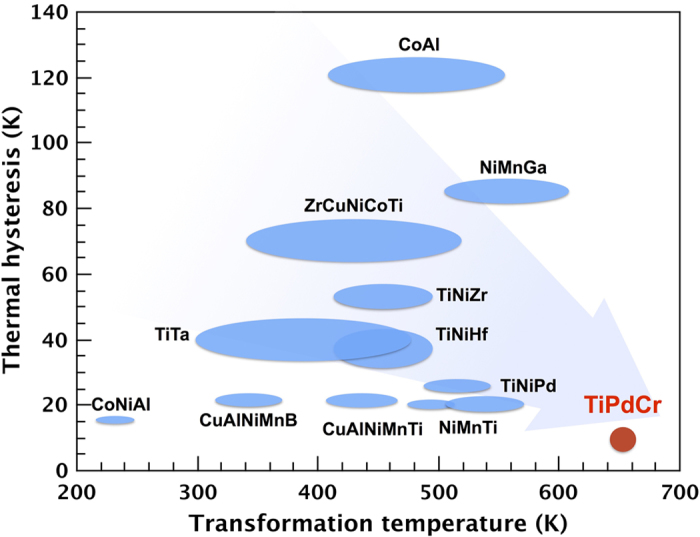
A comparison of the thermal hysteresis and transformation temperature for the bulk SMAs. The thermal hysteresis and transformation temperature values of CoAl^27^, ZrCuNiCoTi^28^, NiMnGa^29^,^30^, TiNiHf^31^, TiNiPd^32^,^33^, TiNiZr^34^,^35^, TiTa^36^, CoNiAl^37^, ZnAuCu^3^, CuAlNiMnB^38^, TiNiCuPd^14^, CuAlNiMnTi^26^, and NiMnTi^39^ are collected from literatures.

Our new alloy does not only have a very small thermal hysteresis, it also possesses a very high transition temperature. Figure 6 compares our results with those reported previously, which is beneficial in the search for high temperature SMAs with small hysteresis and high reversibility^3^,^14^,^26^. We observe that the Au-Cu-Zn and Ti-Ni-Cu-Pd alloys exhibit not only the smallest thermal hysteresis, but also low transformation temperatures. For traditional high temperature SMAs, such as Ti-Ni-Hf, Ti-Ni-Zr, the thermal hysteresis always reaches 30 K or more, indicating poor functional fatigue. Using our design strategy, the Ti_50_Pd_45.3_Cr_4.7_ alloy attains a superior combination of both small thermal hysteresis and high transformation temperature than other high temperature alloys.

In summary, we have proposed a design strategy for finding new SMAs with small thermal hysteresis. That is, a small ΔT can be achieved in the crossover region between two martensitic transformations with opposite changes in electrical resistance at the transformation temperature. By using this, we find that the alloy Ti_50_Pd_45.3_Cr_4.7_ possess both very small thermal hysteresis and high transformation temperature. Moreover, Cr is not the only dopant that can be used to vary Δ*R*. The alloy families Ti-Pd-V, Ti-Pd-Mn, Ti-Pd-Ni are potentially promising systems likely to show similar behavior. Thus, we expect the present design strategy to guide the discovery of new SMAs with small ΔT by either doping or thermal treatment.

## Methods

Base ingots of *Ti*_50_*Pd*_50−*x*_*Cr*_*x*_ (*x* = 4.0, 4.2, 4.4, 4.5, 4.6, 4.7, 4.8, 5.0 *at*%) alloys were made by arc melting a mixture of 99.9% pure Ti, 99.9% pure Pd and 99.9% pure Cr in an argon atmosphere. Specimens for measurement were spark cut from the ingots that were hot rolled to 1 mm thick. They were then solution treated at 1273 K for 1 hour in evacuated quartz tubes and quenched into ice water. In order to remove the affected surface layer, the specimens were mechanically polished, and followed by chemical etching.

A temperature dependent resistance (R(T)) measurement was made with a cooling/heating rate of 2 *Kmin*^−1^ to detect the resistance change (+Δ*R* or −Δ*R*) at the martensitic transformation. Differential scanning calorimetry (DSC) measurements were also employed with a cooling/heating rate of 10 *Kmin*^−1^ to measure the martensitic transformation by exothermal/endothermic peaks.

## Additional Information

**How to cite this article**: Xue, D. *et al*. Design of High Temperature Ti-Pd-Cr Shape Memory Alloys with Small Thermal Hysteresis. *Sci. Rep.* **6**, 28244; doi: 10.1038/srep28244 (2016).
